# Tissue Plasminogen Activator Binding to Superparamagnetic Iron Oxide Nanoparticle—Covalent Versus Adsorptive Approach

**DOI:** 10.1186/s11671-016-1521-7

**Published:** 2016-06-14

**Authors:** Ralf P Friedrich, Jan Zaloga, Eveline Schreiber, Ildikó Y Tóth, Etelka Tombácz, Stefan Lyer, Christoph Alexiou

**Affiliations:** Department of Otorhinolaryngology, Head and Neck, Section for Experimental Oncology and Nanomedicine (SEON), Else Kröner-Fresenius-Stiftung-Professorship, University Hospital Erlangen, Glueckstraße 10a, 91054 Erlangen, Germany; Department of Physical Chemistry and Materials Science, University of Szeged, Szeged, Hungary

**Keywords:** Protein binding, Activated ester reaction, Fibrinolysis, tPA, Tissue plasminogen activator, Iron oxide nanoparticles, Flow cytometry

## Abstract

Functionalized superparamagnetic iron oxide nanoparticles are frequently used to develop vehicles for drug delivery, hyperthermia, and photodynamic therapy and as tools used for magnetic separation and purification of proteins or for biomolecular imaging. Depending on the application, there are various possible covalent and non-covalent approaches for the functionalization of particles, each of them shows different advantages and disadvantages for drug release and activity at the desired location.

Particularly important for the production of adsorptive and covalent bound drugs to nanoparticles is the pureness of the involved formulation. Especially the covalent binding strategy demands defined chemistry of the drug, which is stabilized by excess free amino acids which could reduce reaction efficiency. In this study, we therefore used tangential flow filtration (TFF) method to purify the drugs before the reaction and used the frequently applied and clinically available recombinant tissue plasminogen activator (tPA; Actilyse^®^) as a proof of concept. We then coupled the tPA preparation to polyacrylic acid-*co*-maleic acid (PAM)-coated superparamagnetic iron oxide nanoparticles (SPIONs) using an amino-reactive activated ester reaction and compared these particles to PAM-coated SPIONs with electrostatically adsorbed tPA.

Using dynamic light scattering (DLS) and pH-dependent electrokinetic mobility measurements, we showed that surface properties of the SPIONs were significantly greater affected after activation of the particles compared to the adsorption controls. Different in vitro assays were used to investigate the activity of tPA after coupling to the particles and purification of the ferrofluid. Covalent linkage significantly improves the reactivity and long-term stability of the conjugated SPION-tPA system compared to simple adsorption. In conclusion, we have shown an effective way to produce SPIONs with covalent and non-covalent ultra-filtrated drugs. We showed that using activated ester reaction, immobilization of the protein was significantly better than in adsorptive approaches. Investigation of those functionalized SPIONs revealed diverging attributes, which should be taken into account when developing nanoparticles for different applications.

**Electronic supplementary material:**

The online version of this article (doi:10.1186/s11671-016-1521-7) contains supplementary material, which is available to authorized users.

## Background

The functionalization of nanoparticles is an important process to generate customized tools for a wide variety of biological and therapeutic applications [1–11]. While the core material particularly determines the physical parameters of the particle system, e.g., magnetic and optical properties, the choice of the shell biomolecules, e.g., proteins, DNA, or RNA, especially defines the surface properties, and thus, their biological behavior [12–14]. Meanwhile, there are plenty of possibilities to conjugate these biomolecules to the particle surface using covalent and non-covalent approaches [15]. However, the surface fabrication has to be done very carefully as it is the most critical factor which defines the properties and effects of the particles. In some cases, it might be advisable to produce particles using different techniques to obtain non-covalent and covalent conjugations and compare the results. While adsorptive binding bears the risk of premature drug release and poor reproducibility, covalent binding could possibly influence drug structure and efficacy [16, 17]. Furthermore, it cannot be assumed that loading efficacy is the same in covalent and non-covalent approaches. However, despite numerous published studies describing the production and efficiency of particles for the treatment of diseases, a direct comparison between different conjugation strategies is not always performed.

In this study, we directly compared the binding of a protein commonly used for the treatment of specific diseases of the circulatory system, the most important cause of premature death in Europe [18]. The main emergency treatment drug for ischemic stroke is the tissue plasminogen activator (tPA), a natural enzyme which catalyzes the conversion of plasminogen to plasmin and thereby dissolves clots. This protein suffers from very low bioavailability and therefore has to be administered in relatively high doses, which can cause potentially serious side effects like internal bleedings. Coupling of the protein to iron oxide nanoparticles for magnetic drug targeting to blood clots could provide higher bioavailability and could help to overcome the drawbacks caused by this high dosage. However, it is also important to ensure that the enzyme can still penetrate into the clot in order to get deep fibrinolysis.

Here, we coupled a clinically available recombinant preparation of tPA (Actilyse^®^) to polyacrylic acid-*co*-maleic acid (PAM)-coated SPIONs using an amino-reactive activated ester reaction. Similar approaches have been taken before [19]; however, the fact that Actilyse^®^ is stabilized by excess arginine which may influence reaction efficacy was not always taken into account. Consequently, the question whether binding of the protein is adsorptive or covalent is still unanswered. Therefore, we first developed a tangential flow filtration (TFF) method, a highly effective purification method for lyophobic colloids [20], to quickly purify the protein of any amino group-bearing by-products which might compromise reaction efficacy. We then covalently attached the protein to the particles and compared the particles with adsorption under the same conditions. We clearly demonstrated the reaction efficacy and the concentration and reaction time-dependent influence on tPA binding on the hydrodynamic size and surface characteristics of the particles using DLS and pH-dependent electrokinetic mobility assays. Although the particles are able to bind a certain amount of protein without further activation, covalent linkage significantly improves the reactivity of the conjugated SPION-tPA system compared to simple adsorption. This leads to tPA-concentration and reaction time-dependent changes in the surface properties, which is reflected by coinciding changes in colloidal stability. We furthermore used different in vitro assays to prove the activity of the protein after particle binding and showed that tPA is still active after coupling to the particles. Particles with covalently bound tPA were significantly more effective than their adsorptive bound counterparts. This strategy can also be used to functionalize nanoparticles with other proteins to target other diseased tissues.

## Methods

### Materials

Iron(II) chloride tetrahydrate (FeCl_2_⋅4H_2_O), hydroxylammonium chloride, bovine serum albumin (BSA), and Eppendorf ultrafiltration tubes with a molecular weight cutoff (MWCO) of 3 kDa were purchased from Merck (Darmstadt, Germany). Iron(III) chloride hexahydrate (FeCl_3_⋅6H_2_O), dialysis tubes (Spectrapor 6, MWCO 8 kDa), ammonium chloride, formic acid, hydrochloric acid 25 %, ethylcarbodiimide hydrochloride (EDC), *N*-hydroxysuccinimide (NHS), sodium thiosulfate, silver nitrate, sodium carbonate, and ammonia solution 25 % were supplied by Roth (Karlsruhe, Germany). Propidium iodide (PI), sodium citrate, triton X-100, ninhydrin, MES, HEPES, and poly(acrylic acid-co-maleic acid) solution (Mw = 3000 Da) were purchased from Sigma-Aldrich (St Louis, MO, USA). Ringer’s solution was bought from Baxter Healthcare (Zurich, Switzerland). Annexin V-FITC (AxV), Hoechst 33342 (Hoe), and DiIC1(5) (hexamethylindodicarbocyanine iodide dye [DiI]) were purchased from Thermo Fisher Scientific (Waltham, MA, USA).

### Synthesis of Nanoparticles

#### Synthesis of In Situ-Coated Poly(acrylic acid-co-maleic acid) Ferrofluid (PAM-SPIONs)

Poly(acrylic acid-co-maleic acid)-coated superparamagnetic iron oxide nanoparticles (PAM-SPIONs) were synthesized using a previously reported protocol [21]. Briefly, iron oxide nanoparticles were synthesized by co-precipitation of iron(II) and iron(III) at a stoichiometric ratio of 1:2 under argon atmosphere at 80 °C. After purification by dialysis, the particles were coated with PAM. The particles were therefore diluted in a 25 % (*w*/*V*) solution of PAM to a total iron concentration of 5 g/L. The pH was adjusted to pH 6.5 using sodium hydroxide solution (5.0 N), and the suspension was stirred for 24 h. Excess polymer was removed by multiple centrifugation and resuspension in ultrapure water.

#### Purification of the tPA Formulation

In order to remove the arginine excess from the tPA formulation, we established a TFF method. The protein alone is not stable in water or isotonic salt solutions. Consequently, early attempts using dialysis or diafiltration were not successful. In this study, we used a Spectrum Labs KrosFlo^®^ Research II TFF unit (SpectrumLabs, Los Angeles, California) with modified polyethersulfone (mPES) hollow fibre filters (molecular weight cut off 30 kDa). After dissolving the lyophilised tPA in 0.3 M HEPES buffer (pH 7.4) (1 mg/mL), the solution was filtered using 0.3 M HEPES buffer (pH 7.4) as filtration medium. The removal of free amino acids was shown using ninhydrin reaction from the filtrate samples. After 20 min of filtration with a constant filtration gradient of 1 mL/min, no free amino acids were detected in the filtrate. The protein solution was then concentrated to the original volume and stored at 4 °C.

#### Synthesis of PAM/Tissue Plasminogen Activator Hybrid Nanoparticles (tPA@PAM-SPIONs)

PAM-SPIONs were diluted to a concentration of 1.0 mg/mL total iron in MES buffer 0.05 M pH 6.3 at 4 °C. 19.9 mg/mL EDC-HCl and 24.0 mg/mL NHS per mL were then added to activate the carboxyl groups at 4 °C in the dark for 1 h [22]. The particles were then centrifuged twice to remove excess activator and resuspended in 0.1 M MES buffer pH 6.3. The adsorption control was processed accordingly (buffers and centrifugation), but no EDC or NHS was added. The respective amount of protein (between 25 and 500 μg tPA per mL, reflecting a tPA to iron weight ratio of 0.3–6.25 %) was then added to the activated or non-activated particles. The mixture was stirred for 3 h at 4 °C. The particles were centrifuged and resuspended in ultrapure water two times and then resuspended in the original volume of ultrapure water. The supernatants of the washing steps were collected, lyophilized, and redissolved in 500 μL water for determination of unbound tPA. The amount of unbound tPA was determined using Coomassie and silver staining, respectively. In short, 25 μL samples and TPA standard, respectively, were denatured in 6× SDS-PAGE sample loading buffer (G-Biosciences, USA) at 95 °C for 5 min before being separated electrophoretically with a 12.5 % SDS polyacrylamide gel at 30 mA for 2 h. After staining with Commassie staining solution (0.5 % (*w*/*V*), Coomassie Brilliant Blue G250 (Biorad), 50 % ethanol, 7 % acidic acid) for 2 h and destaining with 20 % methanol/7 % acidic acid, the gels were scanned and protein bands were quantified using image J, and for silver staining, SDS polyacrylamide gels were incubated in fixer solution (40 % ethanol, 10 % acetic acid) for 30 min, washed twice in 30 % ethanol, and twice with water. Gels were incubated in sensitizer solution (0.02 % Na_2_S_2_O_3_) for 1 min, washed with water, and incubated with silver nitrate solution (100 mM AgNO_3_, 0.02 % formaldehyde) for 20 min, washed with water, and developed (2 % Na_2_CO_3_, 0.04 % formaldehyde) until staining is sufficient. Subsequently, gels were washed with water, scanned, and quantified using image J.

### Characterization of Nanoparticles

#### Nanoparticle Quantification

The total iron content was determined using an Agilent 4200 microwave plasma-atomic emission spectrometer (MP-AES; Agilent Technologies, Santa Clara, CA). Three different aliquots (50 μL) of the respective ferrofluid were dissolved in 950 μL of hydrochloric acid 25 %. The iron content was then determined with MP-AES using an iron solution as external standard. The calibration curve was prepared with iron concentrations ranging from 0.05 to 2.5 mg/L. If the coefficient of determination (*R*^2^) in this given range exceeded 0.999, we proceeded with the sample measurements, which were performed in triplicates.

#### Dynamic Light Scattering (DLS) and Zeta Potential Measurements

Size measurements were performed with a Nanophox (Sympatec, Clausthal-Zellerfeld, Germany) photon cross-correlation spectrometer. The respective suspensions were diluted to a total iron concentration of 25 μg/mL with ultrapure water. They were measured precisely 60 s after sonication in triplicate at 25 °C. The hydrodynamic sizes and zeta potentials in cell culture media were determined using a Malvern Zetasizer Nano ZS (Malvern Instruments, Worcestershire, England). The particles were diluted to a total iron concentration of 25 μg/mL with RPMI 1640 cell culture (without FCS) and measured in triplicate at 25 °C. pH-dependent electrokinetic mobility measurements were also done with the Zetasizer. Here, the particle suspension was diluted to 25 μg/mL total iron in 10 mM sodium chloride solution which had been adjusted to the respective pH using either hydrochloric acid or sodium hydroxide solution. The particles were measured precisely 60 s after sonication.

#### Fourier Transform Infrared Spectroscopy (FTIR)

Five hundred microliters of the respective particles were lyophilized overnight. Spectra of the respective samples were then taken with a BRUKER Alpha FTIR spectrometer (Bruker, Billerica, MA, USA) operated in attenuated total reflection mode from 4000 to 400 cm^−1^ using a step size of 0.5 cm^−1^.

#### Determination of tPA Activity with S-2288

The amidolytic activity of tPA was measured spectroscopically using the chromogenic substrate S-2288™ (Chromogenix, Nr. 8205239, USA). Freshly prepared tPA stock solution (1 mg/mL in water; Actilyse Cathflo, Boehringer Ingelheim, Germany) was diluted to 0.1 mg_tPA_/mL. The tPA working solution (0.1 mg_tPA_/mL) were diluted 1:20, 1:16.6, and 1:13.3, respectively. SPION stock solutions (3 mg_Fe_/mL) and the supernatants of the SPION preparations were diluted to 1:16.6 and 1:1.6, respectively using sterile deionized H_2_O. Fifty microliters of diluted tPA and SPION samples were pipetted in quadruplicates into a 96-well cell culture plate mixed with 100 μL assay buffer (1 mM S-2288 in 0.1 M Tris-HCl pH 8.4). The kinetic of the reaction were recorded by measuring the absorption at 405 nm in a spectrophotometer (FilterMax F5, Molecular Devices, Sunnyvale, CA, USA) overnight. To compare SPION-containing and SPION-free samples, the data of SPION-containing samples were subtracted with the value given by the difference in the starting absorption of samples with SPIONs and control samples.

#### Determination of tPA Activity with Fibrin-Agarose Gels

The activity of tPA was determined by fibrin clot lysis assay using fibrin-containing agarose plates [23, 24]. The plates were prepared by mixing 25 mL agarose solution (3 % in TBS; agarose low melt, # 6351, Carl Roth GmbH, Germany) containing 5 U thrombin (thrombin, # T7009-100 U; dissolved in 1 mL distilled H_2_O; Sigma-Aldrich, USA) with 12.5 mL fibrinogen solution (3 mg/mL in TBS; fibrinogen, F3879; Sigma-Aldrich, USA) at 37 °C. The reaction mix was poured into a 1-well cell culture plate (Greiner Bio-One, Cellstar OneWell Plate, # 670180) equipped with a 24 pin tool with a pin diameter of 3 mm and incubated at 37 °C for 2 h to allow fibrin clot formation, followed by cooling at 4 °C for 1 h. After carefully removing the pin tool, the fibrin-agarose gel with the emerging holes were filled with 20 μL sample and incubated at 37 °C overnight. The degree of clot lysis was estimated by comparing the size of the fibrinolysis zone around the samples.

### Cell Culture and Sample Preparation

#### Cell Cultivation

Primary human umbilical vein endothelial cells (HUVECs) were purchased from PromoCell (Heidelberg, Germany). In all experiments, HUVECs pooled from four donors were used at passage 3–5 which corresponds to 12–20 cell divisions. HUVECs were cultivated in enhanced endothelial cell growth medium from PELOBiotech (Planegg, Germany) at 37 °C and 5.0 % CO_2_. For further passaging, trypsinization was performed according to the manufacturer’s instructions.

#### Cell-Based Experiment Preparation

3.2 × 10^5^ HUVECs were seeded into 25 cm^2^ cell culture plates. After 24 h, SPIONs (PAM-SPIONs, covalent tPA@PAM-SPIONs, and adsorbed tPA@PAM-SPIONs) were added to a final concentration of 30 μg_Fe_/mL cell culture media which corresponds to 7.2 μg_Fe_/cm^2^ cell culture plate area. Thus, the correlation between the SPION concentration in cell culture media and on the plate surface area was kept constant for all experiments. Cells were incubated with SPIONs for another 48 h followed by harvesting. The cell pellets were washed twice with PBS and resuspended in 0.5 mL PBS. Cell suspensions were used to determine the absolute cell counts and viability with the MUSE^®^ Cell Analyzer (Merck-Millipore, Billerica, USA), as well as for flow cytometry analysis and SPION quantification measurements using AES spectroscopy (see below).

#### In Vitro Toxicity on Human Umbilical Vein Endothelial Cells (HUVECs)

##### Analysis of HUVECs Using Flow Cytometry

Flow cytometry was performed using a Gallios cytofluorometer (Beckman Coulter, Fullerton, CA, USA) in order to analyze cell morphology (FSc and SSc) and cell viability [25, 26]. For cell death analysis, 50 μL aliquots of cell suspension were incubated with 250 μL of freshly prepared staining solution containing 1 μg/mL annexin V-FITC, 1 μg/mL Hoechst 33342, and 5.1 μg/mL 1,1′,3,3,3′,3′-hexamethylindodicarbocyanine iodide (DiIC1(5)) (all from Thermo Fisher Scientific, Waltham, MA, USA), 20 μg/mL propidium iodide (Sigma-Aldrich, Taufkirchen, Germany) in Ringer’s solution (Baxter Healthcare, Zurich, Switzerland) for 20 min at 4 °C.

SSc was extracted from the flow cytometric measurements after gating on phenotypically healthy cells, characterized by annexin V negative and propidium iodide negative staining. Every sample was measured for a fixed time (120 s).

For the analysis of cell cycle and DNA degradation, further 200 μL of the cell suspensions were fixed by adding 3 mL of 70 % (*v*/*v*) ice-cold ethanol and stored at −20 °C for further processing. The cells were then centrifuged (5 min, 1000*g*, 4 °C), the supernatant was removed, and the cells were washed with PBS once. Then the cells were resuspended in 0.5 mL PBS, and 0.5 mL DNA extraction buffer (192 mL of 0.2 M Na_2_HPO_4_, 8 mL 0.1 % Triton X-100 (*v*/*v*), pH 7.8) was added and incubated for 5 min at RT. Cells were centrifuged (5 min, 1000*g*, 4 °C), the supernatant was removed, and cells were resuspended in 1 mL PIT DNA staining solution (50 μg/mL propidium iodide in water and 1 mg/mL sodium citrate, 0.1 % Triton X-100 (*v*/*v*),) and incubated for 30 min in the dark. Electronic compensation was used to eliminate bleed through fluorescence. Data analysis was performed with Kaluza software version 2.0 (Beckman Coulter). All flow cytometry analyses were performed in quadruplicates.

#### Quantification of Cellular SPIONs by Atomic Emission Spectroscopy (AES)

The absolute iron content was determined by microwave plasma-atomic emission spectrometer (MP-AES) using an Agilent 4200 MP-AES (Agilent Technologies, Santa Clara, CA). Cell suspensions with 2 × 10^5^ cells were centrifuged for 5 min at 500*g*. The supernatants were aspirated and the pellets stored at −20 °C until measurement. The cell pellets were then dissolved in 0.5 mL nitric acid 65 % overnight and diluted to 4 mL with ultrapure water. The total iron content of the cells was then determined using the MP-AES method which was described above. The iron content was normalized to the total number of cells in the analyzed cell pellet.

## Results

### Production of tPA-Containing Nanoparticles

#### Influence of Solvent and Ultrafiltration of tPA Solutions on tPA Activity

In order to couple proteins to polyacrylic acid-*co*-maleic acid (PAM)-coated SPIONs using an amino-reactive ester reaction, the protein solution should be free of other compounds with reactive NH_2_-groups. The commercially available tPA formulation Actilyse^®^ contains 38.5 mg/mL of the amino acid arginine. Arginine bears at least two free primary amine groups which could possibly react with the activated *o*-acylisourea esters during the EDC/NHS coupling. This could impede the reaction efficacy of tPA to the particle surface. Therefore, we purified the formulations before usage.

To remove the excess of inactive substances, we established a TFF method using a Spectrum Labs KrosFlo^®^ Research II TFF unit with modified polyethersulfone (mPES) hollow fibre filters. HEPES buffer 0.3 M proved to be one of the few solvents in which the protein was sufficiently stable during filtration. We compared the activity of stabilized tPA, tPA dissolved in HEPES filtration buffer and ultrafiltrated tPA in HEPES filtration buffer (Additional file 1: Figure S1). The tPA activity was highest when dissolving the lyophilized protein in water as described by the manufacturer. Changing the solvent to HEPES reduces the activity to 80 % which is further reduced by the ultrafiltration process to 49 %.

#### Production of Covalent and Non-covalent Functionalized SPIONs

**Fig. 1:**
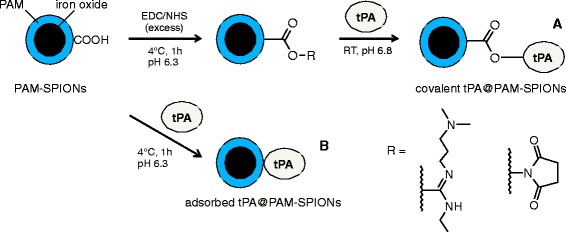
Production of tPA-coated and poly(acrylic acid-*co*-maleic acid) (PAM)-bearing superparamagnetic iron oxide nanoparticles (tPA@PAM-SPIONs)*.* Reaction *A*: SPIONs with covalently bound tPA after EDC/NHC induced activation of PAM-containing carboxyl groups: covalent tPA@PAM-SPIONs. Reaction *B*: SPIONs with adsorbed tPA on the PAM-coated surface: adsorbed tPA@PAM-SPIONs

A simplified reaction scheme of our approach to covalently immobilize tPA on the PAM-coated SPIONs is displayed in Fig. 1. EDC/NHS coupling of proteins to carboxylated surfaces is a well-studied reaction [22]. Due to their high surface-to-volume ratio and surface curvature, nanoparticles often exhibit a different chemistry as far as reaction parameters are concerned [27]. It is therefore important to evaluate reaction parameters case-by-case. Grafting of molecules to SPION surfaces can influence their surface properties and, consequently, their stability. Measurements of hydrodynamic size at a constant time point after redispersion can provide information about changes in stability in tandem with pH-dependent electrokinetic mobility measurements [21]. In this study, we used this method to demonstrate the dependence of colloidal parameters on the reaction conditions after binding of tPA.

#### Differences in tPA-Binding Efficiencies on Covalent and Non-covalent Functionalized SPIONs

**Fig. 2:**
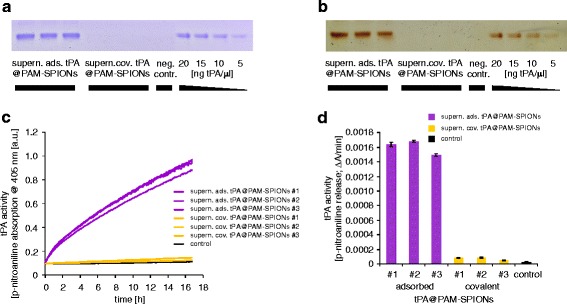
Determination of the tPA binding efficiencies on SPIONs by measurement of the remaining tPA activity in the supernatant of the reactions. **a**, **b** Coomassie and silver staining after SDS-PAGE of the supernatants. **c**, **d** Remaining tPA activity was measured with the chromogenic S-2288 activity assay. **c** Kinetic of the reaction supernatant of covalent and non-covalent functionalized SPIONs determined by the hydrolyzation of S-2288 and the arising p-nitroaniline absorption. **d** tPA activity calculated by the absorption change of p-nitroaniline emerging within the first 2 h during the kinetics shown in (**c**)

We estimated the tPA binding efficiency of three independent covalent and non-covalent reactions by determining the remaining tPA protein and tPA activity in the collected supernatant of the reaction mix and the washing steps (Fig. 2). The total tPA protein content in the supernatant were analyzed by Coomassie and silver staining after SDS-PAGE (Fig. 2a, b). In contrast to the supernatant which were collected during the covalent reaction, the supernatant of the adsorption contained detectable amounts of tPA, indicating a significantly higher tPA load on particles after the covalent reaction (Fig. 2a, b). Quantification of the SDS-PAGE using image J revealed an adsorption of 47.7 ± 5.4 % of the applied tPA, whereas 98.6 ± 0.8 % was bound to the SPIONs after covalent reaction. We confirmed the different binding efficiencies indirectly by measurement of the tPA activity in the supernatant using the S-2288 activity assay (Fig. 2c, d). Only a minimal activity was left in the supernatant of the covalently functionalized particles, indicating that almost all tPA, added to the activated reaction mixes, were bound to the particles. In contrast, the supernatant of the adsorption reactions still exhibits clear enzymatic activity, which was approximately 21.4-fold higher than the supernatant of the covalent reaction mixes. This indicates a superior protein binding rate to nanoparticles with activated carboxyl groups.

### Physico-chemical Characterization of Functionalized Nanoparticles

**Fig. 3:**
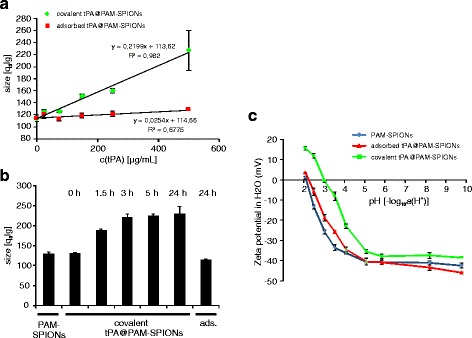
Physical properties of tPA particles. **a** Hydrodynamic diameter of covalent functionalized SPIONs measured at *t* = 60 s after ultrasonication correlates with the used tPA amount. Size increase is significantly higher (*p* < 0.005) for activated particles. **b** Hydrodynamic diameter of covalently functionalized SPIONs (c(tPA) = 500 μg/mL) is dependent on the reaction time (DLS). **c** Grafting of tPA significantly influences surface properties of PAM-SPIONs at c(tPA) = 250 μg/mL

We then evaluated the influence of tPA concentration and reaction time on the PAM-SPIONs using dynamic light scattering (DLS) (Fig. 3). The hydrodynamic particle size of the reacted tPA@PAM-SPIONs which was measured at constant time points after sonication (60 s) increases with good correlation to the tPA amount. The adsorption control does not show such behavior. As the size increase is a direct consequence of a change in surface properties, this could indicate successful surface modification after 3 h of reaction time (Fig. 3a). The high standard deviations for the samples reacted with 500 μg/mL tPA already indicate rapid sedimentation. Figure 3b shows that this effect is also reaction-time dependent. The tPA concentration which was used here was 500 μg/mL. Seemingly, this effect is only taking place during the first 3 h of the reaction. To investigate the changes in the surface properties, we performed pH-dependent electrokinetic mobility measurements on tPA@PAM-SPIONs which reacted or adsorbed for 3 h with 250 μg/mL of tPA (Fig. 3c). Such measurements allow comparison of the surface properties of colloids [21, 28]. The PAM-coated SPIONs display a negative surface charge which begins to decrease at pH 4 and below until reaching the isoelectric point (IEP) at around pH 2. This is in concordance with previous results [21]. tPA addition leads to a shift in the IEP towards pH 3. As the isoelectric point of tPA is about 7.7, this could indicate a successful surface binding of the protein [29]. Furthermore, at pH 2, tPA@PAM-SPIONs bear a positive surface charge of 15.52 ± 0.83 mV, which is not possible for PAM polymer.

Interestingly, in Fourier transform infrared spectroscopy (FTIR), the tPA could not be detected in tPA@PAM-SPIONs even at tPA loading concentrations of 500 μg/mL (Additional file 1: Figure S2). We attribute this to the low molar ratio of tPA relative to iron or polymer.

Nonetheless, taken together, these measurements clearly show the different nature of covalently bound and adsorbed tPA protein on the surface of PAM-SPIONs.

### tPA Activity of Functionalized SPIONs with Covalent and Non-covalent tPA

**Fig. 4:**
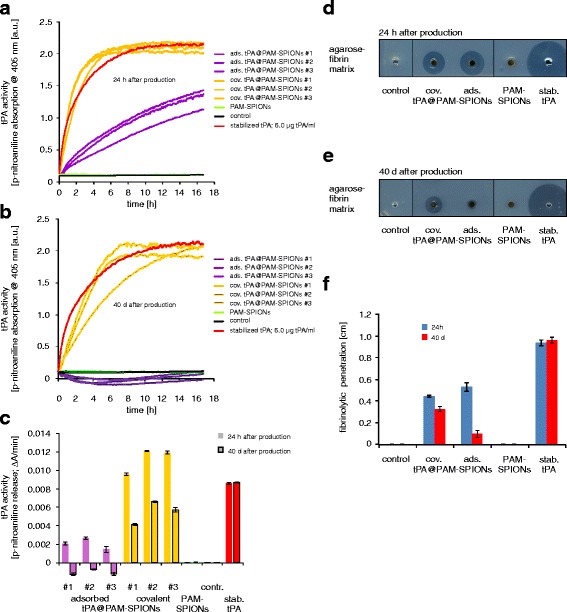
tPA activity of functionalized SPIONs with covalent and non-covalent tPA. **a–c** tPA activity of covalent and non-covalent functionalized SPIONs measured with the chromogenic S-2288 activity assay. **a** tPA activity measured after 24 h. **b** tPA activity measured after 40 days. **c** tPA activity calculated by the absorption change of p-nitroaniline emerging within the first 2 h during the kinetics shown in (**a** and **b**). **d**, **e** Activity measurement of covalent and non-covalent functionalized SPIONs after 24 h and 40 days, measured with thrombus-mimicking fibrin-containing agarose plates. **f** tPA activity calculated by the distance between the wall of the sample holes and the edge of the fibrinolysis zones of no-coated and coated SPIONs after 24 h and 40 days

The decision for a non-covalent or covalent approach is highly dependent of the expected activity of the attached drug at a specific location. Adsorbed proteins might be of advantage because they quickly release the drug at a particular site and with higher penetration through tissues. Covalent strategies might be more effective using drugs that covalently bound to particles, e.g., when the drug must act exclusively at a single location or must be forced into other matrices. Here, we investigated the activity of freshly manufactured particles in solution and in fibrin matrices and compared the results with the activity measured after long time storage at 4 °C (Fig. 4).

We first determined the activity of three independently and freshly manufactured particles with covalent and non-covalent attached tPA using the S-2288 assay (Fig. 4a, c). As expected from the results achieved with the supernatant of the reaction mixes (Fig. 2), covalently bound particles possessed strong enzymatic activity, even stronger than a pure 0.1 mg/mL stabilized tPA control. Considering the dilution of the samples, the tPA activity of particles with covalently bound tPA is in average 40 % higher than the activity of a 0.1 mg/mL stabilized tPA solution. In contrast, the adsorptive approaches deliver particles with a tPA activity of 22 % if compared to the tPA control (Fig. 4a, c). Hence, the activity of SPIONs with covalently bound tPA is 6.4-fold higher than the non-covalent approach of tPA binding.

To investigate the effects of long time storage, we kept the particles at 4 °C and repeated the experiments after 40 days (Fig. 4b, c). Remarkably, the particles with covalently bound tPA still exhibit 63 % activity compared to the tPA control samples, whereas the SPIONs with adsorbed tPA were absent of any remaining tPA activity as measured by the S-2288 assay, indicating an effective stabilization of tPA when covalently bound to a matrix.

We then used a fibrin-agarose assay to investigate the behavior of these SPIONs within a matrix and to evaluate the fibrinolytic activity of the functionalized SPIONs (Fig. 4d, f). Both particle systems showed the ability to diffuse into the fibrin-agarose matrix and lysate fibrin fibers. After 24 h, the lysis distance induced by adsorbed tPA@PAM-SPIONs was 19 % larger than those induced by covalent tPA@PAM-SPIONs, despite higher tPA loading and activity of covalent tPA@PAM-SPIONs (Fig. 4d, f). This may already indicate better liberation of tPA from the particles, which may also be beneficial in thrombolysis. Most likely, adsorbed tPA@PAM-SPIONs release small quantities tPA with a considerably higher matrix-diffusion capability. The results of the fibrin-agarose assay significantly changed with aged particles (Fig. 4e, f). While covalent tPA@PAM-SPIONs still shows 74 % of its original activity, the distance of the fibrinolysis zone of adsorbed tPA@PAM-SPIONs dropped to 18 %, confirming the findings of the S-2288 assays.

### Toxicity and Cellular Uptake of tPA-Containing Nanoparticles

Finally, we performed toxicity and cellular uptake experiments to examine possible differences in the cellular response to covalent and non-covalent particles. Human umbilical vein endothelial cells (HUVECs) were incubated with 30 μg_Fe_/mL SPIONs for 48 h. After cell harvesting and counting using the MUSE cell analyzer, toxicity and content of intracellular and cell-associated SPIONs were determined by flow cytometry and microwave plasma-atomic emission spectrometer (MP-AES), respectively (Additional file 1: Figure S3).

Flow cytometry is an accurate technology to analyze cell morphology and cell viability [26, 30–35]. To find possible differences between covalent and non-covalent binding approaches, we performed a multiparameter flow cytometry assay (Additional file 1: Figure S3A–D). After incubation of HUVECs with different particles, a detailed analysis of the membrane potential by DiIC1(5) staining and the viability by annexin V and propidium iodide staining revealed only very slight, non-significant cellular effects compared with control samples (Additional file 1: Figure S3A, B). Merely the propidium iodide-TritonX measurements for the investigation of cell cycle and DNA degradation showed a slightly enhanced DNA degradation in SPION-treated samples, compared to control samples (Additional file 1: Figure S3 C). However, differences between tPA-free and tPA-containing SPIONs were not detectable with the examined parameters, indicating that HUVECs only respond to the SPION-load and were unable to differentiate between tPA-loaded or unloaded SPIONs, given that the cellular interaction with the applied SPIONs is equal. We therefore evaluated the cellular SPION load of the obtained cell suspensions by flow cytometry, as the amount of cellular uptake has been shown to correlate with the flow cytometry analysis of the side scatter [25]. As expected, the determination of the side scatter revealed very similar results of cells incubated with the employed particle systems (Additional file 1: Figure S3D). To confirm these findings, we investigated aliquots of the cell suspension with MP-AES (Additional file 1: Figure S3E). After normalizing the iron content to the cell numbers, we found no significant differences between the cellular uptake of PAM-SPIONs, covalent tPA@PAM-SPIONs and adsorbed tPA@PAm-SPIONs, resembling the data received from flow cytometry and indicating that the tPA shell is not influencing the uptake of particles into HUVECs.

## Discussion and Conclusion

Iron oxide nanoparticles are frequently used within medicine, biotechnology, and catalysis. While the core material particularly determines the physical properties such as size, crystal structure, and mainly magnetic features, the shell is primarily responsible for the biological function of the particle system besides its inherent role in inhibiting particle aggregation [13, 14, 21, 36]. The functionalization of particles with biologically active compounds can be achieved by covalent or non-covalent binding strategy. Since the conjugation of drugs can affect the particle surface, it can lead to deviant behavior between particles with covalently linked and adsorptive bound drugs. Consequently, this influences the in vitro and in vivo behavior, biocompatibility, stability, and solubility and needs to be considered early during the design of new nanoparticles [15, 21]. Moreover, depending on the intended application, the two approaches might have both advantages and disadvantages and have to be weighed very carefully. Often it is difficult if not impossible to predict the properties before manufacturing, making it advisable to produce both versions and to choose the best one after extensive characterization. An elucidatory example is tPA-decorated particles against thrombosis. The ideal tPA-functionalized particle should be chemically and colloidally stable, biocompatible, non-toxic, controllable by an external magnetic field, enzymatically active on fibrin matrices, and display the ability to allow deeper penetration of tPA into fibrin matrices or thrombi.

In this study, we manufactured particles with covalent and non-covalent bound tPA. While the supernatant of ads. tPA@PAM-SPIONs still displayed significant fibrinolytic activity, which we attribute to unbound tPA, the supernatant of cov. tPA@PAM-SPIONs showed no such effect, indicating that covalent binding using an active ester reaction led to higher binding rates. Accordingly, SDS-PAGE determination of supernatants showed free tPA only in samples following adsorbed tPA binding (Fig. 2).

Covalent or non-covalent binding of proteins onto nanoparticles can significantly alter the structure and function of the involved proteins and can in some cases be monitored by FTIR [37–39]. This detection is strongly limited by the signal-to-noise ratio and the mass ratio between nanoparticle and protein. In case of tPA@PAM-SPIONs, the protein is not detectable by FTIR (Additional file 1: Figure S2). However, we were able to show that covalently attached tPA significantly affects the surface chemistry of PAM-SPIONs (Fig. 3c). The IEP of cov. tPA@PAM-SPIONs is clearly shifted towards the IEP of pure tPA compared to PAM-SPIONs. Furthermore, positive zeta potentials can be achieved at low pH. This tPA amount-dependent change in surface charging leads to destabilization of the colloid. Measuring the hydrodynamic size of particle aggregates at constant time points after ultrasonication, we could show that the destabilization depends on the amount of protein used (Fig. 3a) and on the reaction time (Fig. 3b). The surface charging of ads. tPA@PAM-SPIONs is not affected, and therefore, the adsorbed particles show no such instability.

Monitoring the tPA activity of the particles using the S-2288 assay, we showed that the cov. tPA@PAM-SPIONs show significantly higher fibrinolytic activity than the ads. tPA@PAM-SPIONs in vitro (Fig. 4a). Interestingly, the fibrinolytic activity of cov. tPA@PAM-SPIONs was comparable to that of free tPA. This is surprising, because proteins have the tendency to adsorb to surfaces, resulting in a loss of significant amounts of tPA on filters and hose system during the necessary ultrafiltration process and so in a decrease in their measurable activity. We found that these issues result in loss of around 50 % activity after reconstitution in HEPES buffer and ultrafiltration compared to reconstitution in distilled water as shown in Additional file 1: Figure S1. Interestingly, the high difference in tPA activity could not be verified within agarose-fibrin matrices, where the effect of both particle systems were very similar (Fig. 4d). Most likely, the adsorptive bound tPA detaches from the particles and diffuses more readily into the matrix than covalently bound tPA. However, particles with covalently bound tPA seem hypothetically more suitable for magnetic drug targeting, as those particles can be directed to and into a thrombus without stripping off the bioactive tPA enzyme. This topic will be investigated in detail in future studies.

Another issue and one of the most crucial problems of producing tPA-containing medicines is the long term stability. According to the manufacturer, reconstituted tPA solutions are only stable 24 h at 2–8 °C. However, the elapsed time between manufacturing, storage, delivery, and application of tPA particles is significantly longer. Interestingly, tPA conjugated to SiO_2_-magnetic nanoparticles showed an enhanced storage stability up to 9.5-fold in buffer and 2.8-fold in whole blood [24]. To test whether our particles show similar stability, we measured the activity after 40 days to evaluate the long time stability of tPA (Fig. 4b). Impressively, particles with covalently bound tPA still have 49 % of the original activity, whereas the activity of the adsorptive bound tPA is not measurable with the S-2288 assay (Fig. 4c). Similar results were achieved using the agarose-fibrin assay (Fig. 4d–f). The ability of covalent tPA@PAM-SPIONs to penetrate into and dissolve the fibrin network was only reduced by 26 %, while adsorptive tPA@PAM-SPIONs revealed a reduction of 72 % (Fig. 4f).

In summary, the tPA loading efficiency, the enzymatic activity, and the longtime stability of covalent tPA@PAM-SPIONs is much more pronounced than with adsorptive tPA@PAM-SPIONs.

## Abbreviations

AxV, annexin V-FITC; DiIC1(5): hexamethylindodicarbocyanine iodide dye; DLS, dynamic light scattering; EDC, ethylcarbodiimide hydrochloride; FTIR, Fourier transform infrared spectroscopy; HEPES, 4-(2-hydroxyethyl)-1-piperazineethanesulfonic acid; HUVECs, human umbilical vein endothelial cell; IEP, isoelectric point; MP-AES, microwave plasma-atomic emission spectrometer; mPES, modified polyethersulfone; NHS, *N*-hydroxysuccinimide; PAM, polyacrylic acid-*co*-maleic acid; PI, propidium iodide; PIT, propidium iodide/tritonX analysis; SDS-PAGE, sodium dodecyl sulfate polyacrylamide gel electrophoresis; SPIONs, superparamagnetic iron oxide nanoparticles; TFF, tangential flow filtration; tPA, tissue plasminogen activator

## Additional file

Additional file 1: Figure S1. The influence of buffer and diafiltration on the activity of different tPA concentration and dilutions measured with the chromogenic S-2288 activity assay. (A) Kinetic of H2O-diluted tPA and buffer-diluted tPA before and after ultrafiltration determined by the hydrolysation of S-2288 and the arising p-nitroaniline absorption. (B) tPA activity calculated by the absorption change of p-nitroaniline emerging within the first 2 h during the kinetics shown in (A). **Figure S2**: FTIR of particles. Spectra from 4000 to 400cm^−1^ of pure tPA (blue), PAM-SPIONs (red) and covalent tPA@PAM-SPIONs (green). The iron oxide peak at 584 cm^−1^ is clearly visible in the particle containing samples, also the carboxyl peak of PAM can be seen in the polymer containing samples at 1625 cm^−1^. Compared to the PAM-SPION spectrum, there is no concise tPA-derived peak visible within the tPA@PAM-SPIONs spectrum. **Figure S3.** Cellular effects of tPA particles. (A) Membrane integrity analyzed by DiIC1(5) staining. (B) Cell viability determined by annexin V/propidium iodide staining. (PI+) necrotic/late apoptotic cells, (AxV+, PI−) early apoptotic cells, (AxV−, PI−) viable cells. (C) DNA cycle and DNA degradation determined by propidium iodide/tritonX analysis (PIT). The results were normalized to untreated control cells, set to 100 %. (D) Cellular iron uptake in HUVECs determined by flow cytometry analyses of the side scatter. (E) Cellular iron uptake in HUVECs determined by atomic emission spectroscopy (AES). (DOCX 524 kb)
