# Supplementary material for: Tissue Plasminogen Activator Binding to Superparamagnetic Iron Oxide Nanoparticle—Covalent Versus Adsorptive Approach
Source: Nanoscale Res Lett. 2016 Jun 14;11:297. doi: 10.1186/s11671-016-1521-7 (PMC4907967; doi:10.1186/s11671-016-1521-7)
Supplement: Additional file 1: Figure S1. — The influence of buffer and diafiltration on the activity of different tPA concentration and dilutions measured with the chromogenic S-2288 activity assay. (A) Kinetic of H2O-diluted tPA and buffer-diluted tPA before and after ultrafiltration determined by the hydrolysation of S-2288 and the arising p-nitroaniline absorption. (B) tPA activity calculated by the absorption change of p-nitroaniline emerging within the first 2 h during the kinetics shown in (A). Figure S2: FTIR of particles. Spectra from 4000 to 400cm−1 of pure tPA (blue), PAM-SPIONs (red) and covalent tPA@PAM-SPIONs (green). The iron oxide peak at 584 cm−1 is clearly visible in the particle containing samples, also the carboxyl peak of PAM can be seen in the polymer containing samples at 1625 cm−1. Compared to the PAM-SPION spectrum, there is no concise tPA-derived peak visible within the tPA@PAM-SPIONs spectrum. Figure S3. Cellular effects of tPA particles. (A) Membrane integrity analyzed by DiIC1(5) staining. (B) Cell viability determined by annexin V/propidium iodide staining. (PI+) necrotic/late apoptotic cells, (AxV+, PI−) early apoptotic cells, (AxV−, PI−) viable cells. (C) DNA cycle and DNA degradation determined by propidium iodide/tritonX analysis (PIT). The results were normalized to untreated control cells, set to 100 %. (D) Cellular iron uptake in HUVECs determined by flow cytometry analyses of the side scatter. (E) Cellular iron uptake in HUVECs determined by atomic emission spectroscopy (AES). (DOCX 524 kb) [file 11671_2016_1521_MOESM1_ESM.docx]

**Supplementary Material**

**Tissue plasminogen activator binding to superparamagnetic iron oxide nanoparticles – covalent versus adsorptive approach**

Ralf P. Friedrich^1&^, Jan Zaloga^1&^, Eveline Schreiber^1^, Ildikó Y Tóth^2^, Etelka Tombácz^2^, Stefan Lyer^1^, Christoph Alexiou^1*^

*^1^ Department of Otorhinolaryngology, Section for Experimental Oncology and Nano-medicine (SEON), Else Kröner-Fresenius-Stiftung-Professorship, University Hospital Erlangen, Germany. E-Mail: ralf.friedrich@uk-erlangen.de (R.P.F.), jan.zaloga@uk-erlangen.de (J.Z.), eveline.schreiber@uk-erlangen.de (E.S.), stefan.lyer@uk-erlangen.de (S.L.), christoph.alexiou@uk-erlangen.de (C.A.). ^2^ Department of Physical Chemistry and Materials Science, University of Szeged, Hungary. E-Mail:* ildiko.toth@chem.u-szeged.hu *(I.T.), tombacz@chem.u-szeged.hu (E.T.). ^&^ These authors contributed equally to this work.*

**Correspondence**

*Corresponding author: Christoph Alexiou

Department of Otorhinolaryngology, Head and Neck Surgery, Section for Experimental Oncology and Nanomedicine (SEON), Glueckstraße 10a, 91054 Erlangen, Germany

Tel: +49 9131 853 4769

Fax: +49 9131 853 4828

Email: christoph.alexiou@uk-erlangen.de


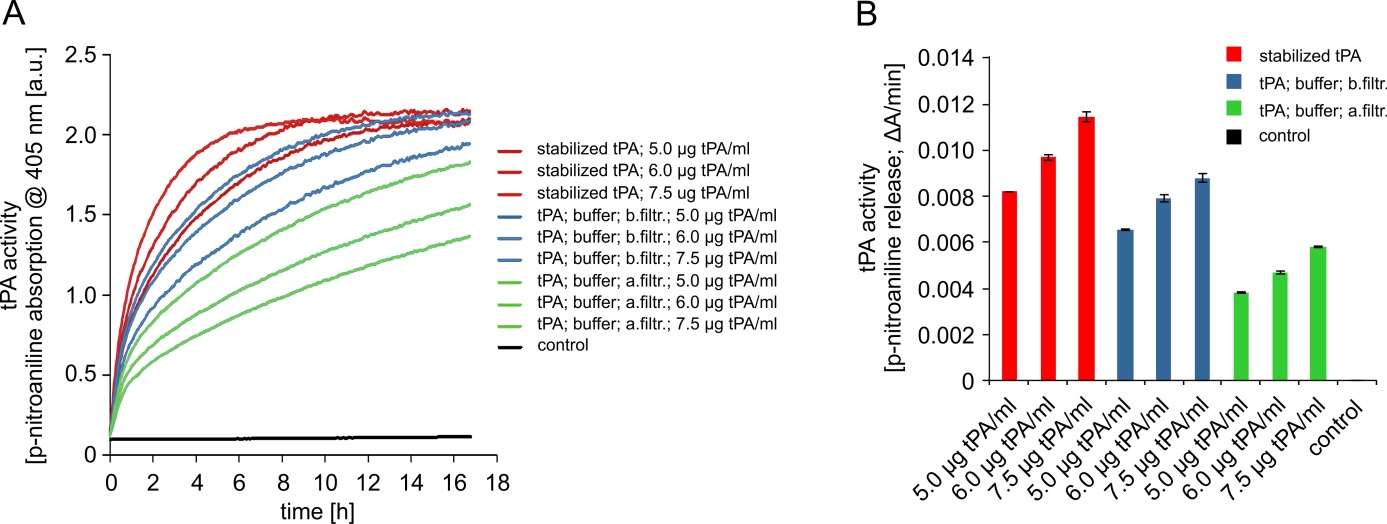


Figure S1: ***Influence of buffer and diafiltration on the activity of different tPA concentration and dilutions measured with the chromogenic S-2288 activity assay***. (A) Kinetic of H_2_O-diluted tPA and buffer-diluted tPA before and after ultrafiltration determined by the hydrolysation of S-2288 and the arising p-nitroaniline absorption. (B) tPA activity calculated by the absorption change of p-nitroaniline emerging within the first two hours during the kinetics shown in (A).

**
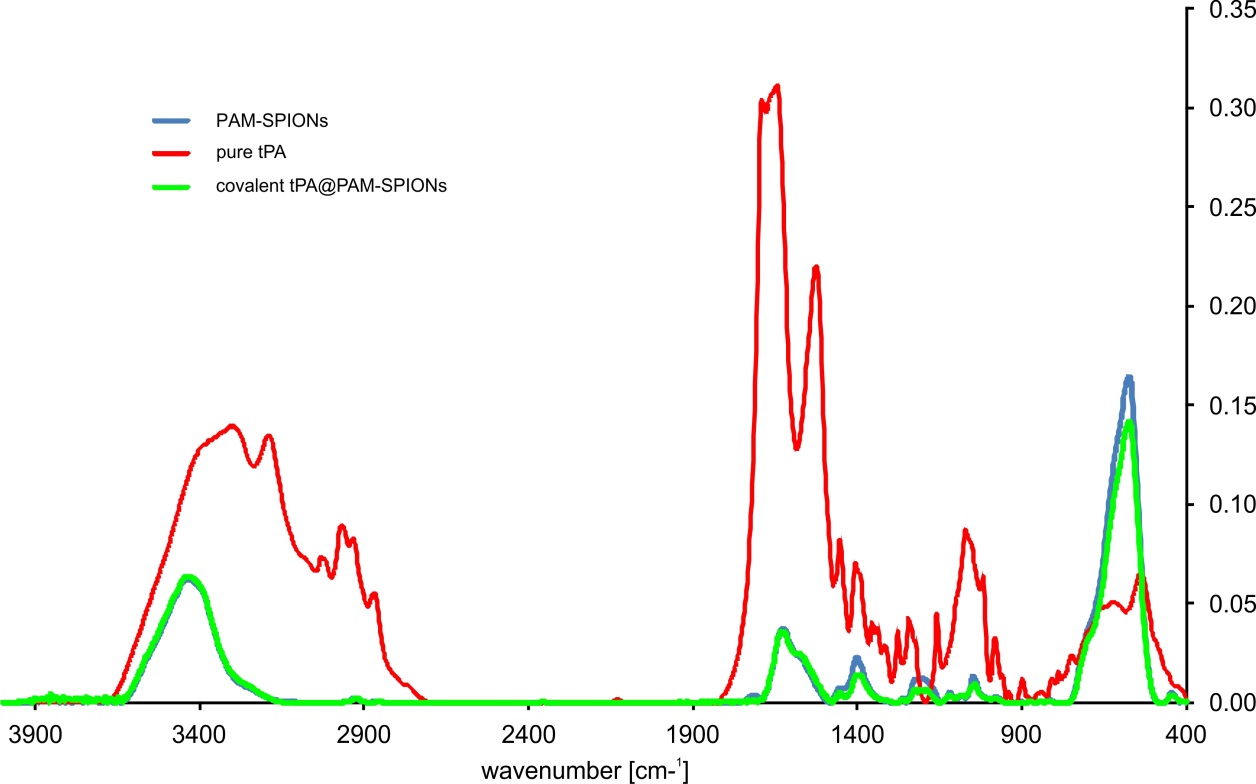
**

Figure S2: ***FTIR of particles***. Spectra from 4000 cm-1 to 400 cm-1 of pure tPA (blue), PAM-SPIONs (red) and covalent tPA@PAM-SPIONs (green). The iron oxide peak at 584 cm-1 is clearly visible in the particle containing samples, also the carboxyl peak of PAM can be seen in the polymer containing samples at 1625 cm-1. Compared to the PAM-SPION spectrum, there is no concise tPA-derived peak visible within the tPA@PAM-SPIONs spectrum.


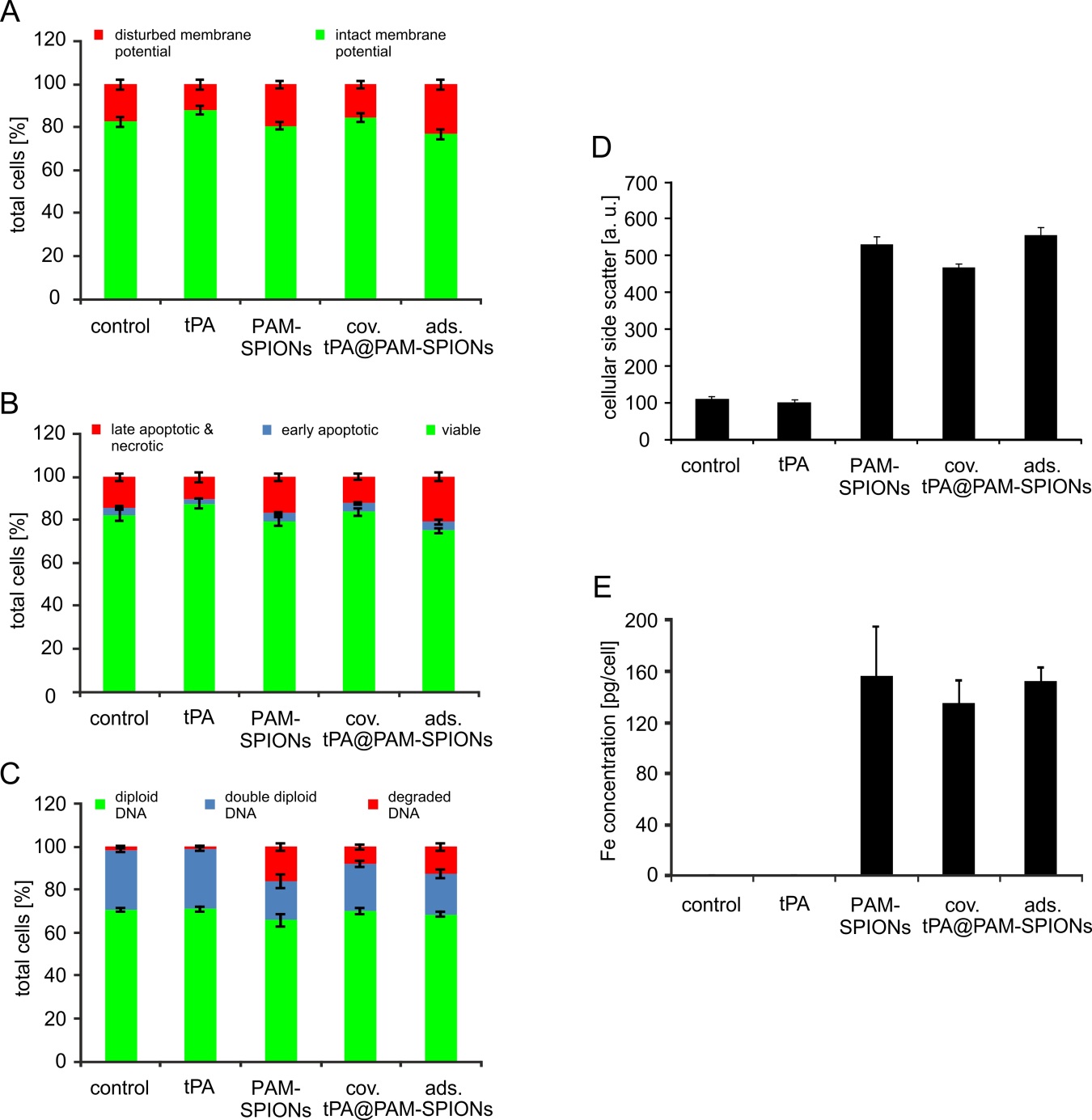


Figure S3. ***Cellular effects of tPA particles*.** (A) Membrane integrity analyzed by DiIC1(5) staining. (B) Cell viability determined by Annexin V/propidium iodide staining. (PI+) necrotic/late apoptotic cells, (AxV+, PI-) early apoptotic cells, (AxV-, PI-) viable cells. (C) DNA cycle and DNA degradation determined by propidium iodide/tritonX analysis (PIT). The results were normalized to untreated control cells, set to 100%. (D) Cellular iron uptake in HUVECs determined by flow cytometry analyses of the side scatter. (E) Cellular iron uptake in HUVECs determined by atomic emission spectroscopy (AES).
